# What is the overall impact or effectiveness of visiting primary health care services in rural and remote communities in high-income countries? A systematic review

**DOI:** 10.1186/s12913-018-3269-5

**Published:** 2018-06-19

**Authors:** Timothy A. Carey, David Sirett, Deborah Russell, John S. Humphreys, John Wakerman

**Affiliations:** 1 Centre for Remote Health, Flinders University, PO Box 4066, Alice Springs, NT 0871 Australia; 2 School of Rural Health, Monash University, Clayton, Australia; 3 Flinders NT, Flinders University, Darwin, Australia

**Keywords:** Primary health care, Visiting services, Impact, Effectiveness, Rural and remote

## Abstract

**Background:**

Visiting services address the problem of workforce deficit and access to effective primary health care services in isolated remote and rural locations. Little is known about their impact or effectiveness and thereby the extent to which they are helping to reduce the disparity in access and health outcomes between people living in remote areas compared with people living in urban regions of Australia. The objective of this study was to answer the question “What is the impact or effectiveness when different types of primary health care services visit, rather than reside in, rural and remote communities?”

**Method:**

We conducted a systematic review of peer-reviewed literature from established databases. We also searched relevant websites for ‘grey’ literature and contacted several key informants to identify other relevant reference material. All papers were reviewed by at least two assessors according to agreed inclusion and exclusion criteria.

**Results:**

Initially, 345 papers were identified and, from this selection, 17 papers were considered relevant for inclusion. Following full paper review, another ten papers were excluded leaving seven papers that provided some information about the impact or effectiveness of visiting services. The papers varied with regard to study design (ranging from cluster randomised controlled trials to a case study), research quality, and the strength of their conclusions. In relation to effectiveness or impact, results were mixed. There was a lack of consistent data regarding the features or characteristics of visiting services that enhance their effectiveness or impact. Almost invariably the evaluations assessed the service provided but only two papers mentioned any aspect of the visiting features within which service provision occurred such as who did the visiting and how often they visited.

**Conclusions:**

There is currently an inadequate evidence base from which to make decisions about the effectiveness of visiting services or how visiting services should be structured in order to achieve better health outcomes for people living in remote and rural areas. Given this knowledge gap, we suggest that more rigorous evaluation of visiting services in meeting community health needs is required, and that evaluation should be guided by a number of salient principles.

## Background

People living in remote locations, particularly in less populated countries such as Australia, experience poorer health compared to people living in cities [1]. Consequently, improving timely access to effective primary health care services to address the health disparity is a government priority. Over recent years, providing primary health care through various forms of visiting services has been a major strategy in Australia to increase access and to improve the health outcomes for remote residents. While visiting services can be configured in a variety of different ways, they generally conform to two basic models [2]. In the first model, an individual or a team operates from a central hub and travels to remote locations on a periodic basis. In the second model, the individual or the team travels in a continuous circuit between different remote locations.

Given the considerable investment required to operate these visiting services, it is imperative to determine how effective they are in reducing the health status inequity between metropolitan, regional, and remote residents. “Effectiveness” in this context is understood broadly to mean any improvement in health conditions or outcomes following the introduction of a service, program, or treatment. To that end, we conducted a systematic review to answer the question “What is the overall impact or effectiveness of visiting primary health care services in rural and remote communities in high-income countries?”. In particular, we were interested in literature that had some relevance to remote health care in Australia.

Visiting, or fly-in/fly out (FIFO), services have been introduced as a strategy to both improve access to health care in remote areas and also to improve the retention of these health care providers [3, 4]. This strategy is based on the premise that remote communities with small populations are unable to support resident health professionals. There are growing concerns, however, about the impact of visiting services on both patients in the community as well as on any existing resident health professionals [5, 6]. In some cases, it appears that FIFO services are used in communities that are, in fact, large enough to support resident health professionals and that, in these situations, the visiting service may be having a “deleterious effect” on the community [7].

The provision of visiting services has not been problem free. For example, there is compelling evidence that, in remote Australian Aboriginal communities, strong relationships are essential to the delivery of effective health services. Characteristic of the relationships which promote effective health services are communication and trust [8–11]. These important relationships, however, take time to develop, so this critical contextual aspect of effective remote primary health care is highly problematic for short-term visiting health professionals.

Social, demographic, and geographic context is a fundamental consideration in ensuring that primary health care is effective and sustainable in remote settings. Visiting health professionals, for example, may not have the knowledge and experience necessary to treat the health conditions that can arise in remote Australia [6, 12]. Anecdotal evidence suggests that this knowledge gap can result in more medical evacuations. There are also important cultural considerations to account for when delivering services in remote Aboriginal and Torres Strait Islander communities [11, 13], and these can be difficult to learn within restricted time-frames.

The coordination of multiple visiting services is also important for the provision of effective health care and this is often difficult in remote settings [6]. For example, when services are not well coordinated the demand for appropriate working spaces can exceed the resources that are available [11]. Furthermore, with multiple visiting health professionals arriving simultaneously, resident staff do not have sufficient time for tasks such as case conferencing and skill development [11].

Internationally, the literature with regard to improving access to health workers is very limited [3]. Similarly, in Australia there is a paucity of comprehensive and reliable data about the impact that the increase in short-term visiting health professionals is having on the effectiveness of remote primary health care services. In order for health service managers, policy-makers, and other decision makers to be able to produce sound, evidence-based decisions, it is vital that the effectiveness or impact (or both) of visiting services, in general, is well understood.

## Method

### Search strategy

Initially, scoping searches of established databases such as Medline and EBSCO were conducted. The search strategy was developed with the assistance of a librarian. Table 1 lists the inclusion and exclusion criteria for the review. It should be noted that, given the very different contexts in high-income countries (HICs) compared to middle-income and low-income countries (LMICs), particularly with regard to available health resources, a decision was made to focus the review on HICs. (A review protocol was not established in the public domain; however, procedures and forms are available from the corresponding author).

**Table 1:** Inclusion and exclusion standards according to specified criteria

Criteria	Inclusion	Exclusion
Time period	1990–2013	Before 1990
Language	English	Other languages
Geographical delimitation	High income economy (World Bank definition)	Low and Middle income countries
Level of health care	Primary Healthcare (WHO definition)	Secondary, tertiary services
Aim: to identify the impact or effectiveness of types of visiting services	• Paper must evaluate the impact or effectiveness (or both) of one or more visiting models	• Paper does not evaluate either impact or effectiveness

Four established databases and a large number of websites were searched (see Table 2). In general, the same terms that were used for the database searches were used when searching websites for the grey literature. Key informants, including members of the research team, also contributed papers for consideration.

**Table 2:** Searches conducted throughout databases and the grey literature

Type of Information	Source	Strategy
Databases	• Ovid Medline • EBSCO CINAHL • Informit • Cochrane Library	(visiting OR outreach OR mobile OR “fly in fly out” OR fifo OR “drive in drive out” OR locum$ OR “hub and spoke”) AND (primary health care OR primary care) AND (rural OR remote) AND (evaluat$ OR efficien$ OR impact OR effective$)
Grey Literature	• Healthinfonet (www.healthinfonet.ecu.edu.au) • National Rural Health Alliance (http://www.ruralhealth.org.au/) • Services for Australian Rural and Remote Allied Health (www.sarrah.org.au) • CRANAplus (https://crana.org.au/) • Health Systems Evidence (http://www.healthsystemsevidence.org) • Australia Institute of Health and Welfare (http://www.aihw.gov.au/) • Health Workforce Australia (http://www.health.gov.au/internet/main/publishing.nsf/Content/hwa-archived-publications) • Northern Territory Government Department of Health (http://www.health.nt.gov.au/) • Western Australian Government Department of Health (http://www.health.wa.gov.au/home/) • South Australian Government Department of Health (http://www.sahealth.sa.gov.au) • Tasmanian Government Department of Health and Human Services (http://www.dhhs.tas.gov.au/) • Victorian Government Department of Health (http://www.health.vic.gov.au/) • New South Wales Government Department of Health (http://www.health.nsw.gov.au) • Queensland Government Department of Health (http://www.health.qld.gov.au/) • Australian Government Department of Health (http://www.health.gov.au/)	The same search terms were used for these websites as was used for the databases.

### Methods of screening and selection criteria

**Fig. 1:**
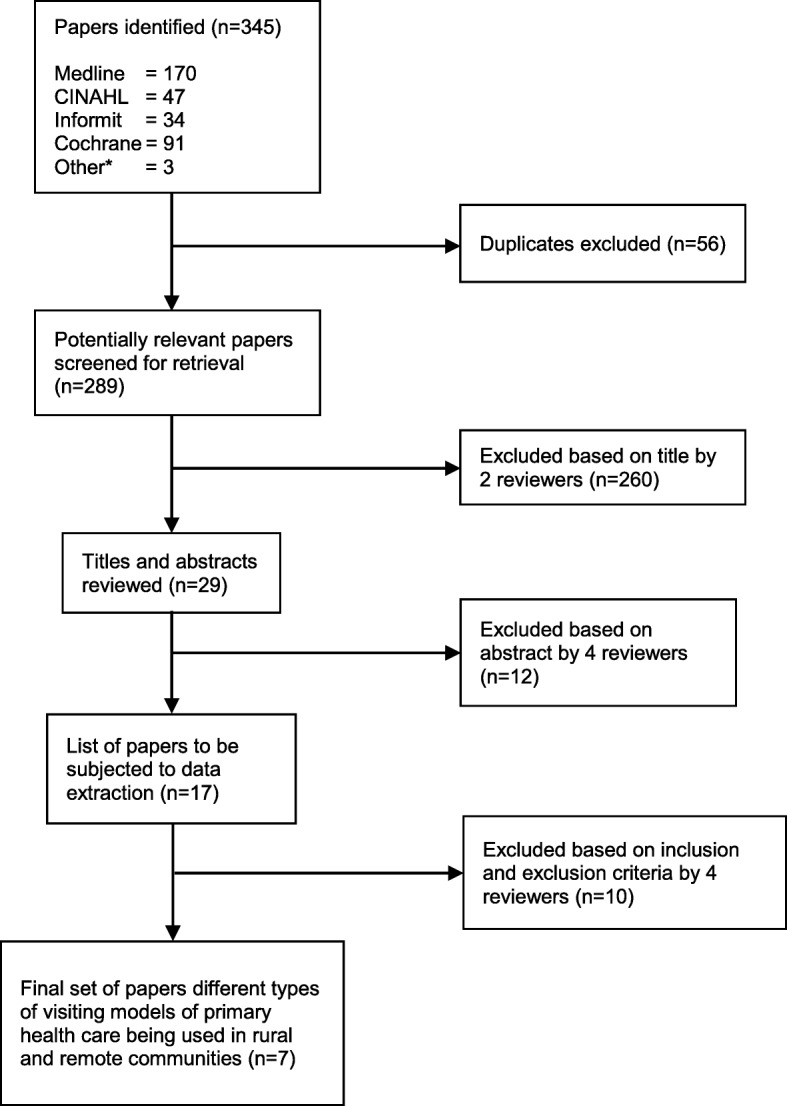
Electronic database selection process. *‘Other’ indicates papers obtained through the grey literature or key informants

Figure 1 illustrates the screening process by which the final set of papers were derived. Once duplicate titles were removed from the initial search, two reviewers scanned the titles from the total pool of titles obtained from the search. Next, titles and abstracts were reviewed by the research team to identify the eligible papers according to the research question and the inclusion and exclusion criteria (see Table 1). From the review of titles and abstracts a small number of papers were retained. These were reviewed by the same team who examined the titles and abstracts using a standard data extraction pro-forma. The data extraction form enabled the researchers to examine the scientific quality of each paper by analysing components such as the objective of enquiry, the methods used, and the design of the study. The data extraction process was completed with the team working in two sets of two researchers. Each set extracted data from half the retained papers. This process led to more papers being excluded. If there were discrepancies between the two reviewers, a fifth researcher adjudicated.

### Data extraction

From the final set of papers, of primary interest were the conclusions that were made regarding service effectiveness or impact. Also of interest were the methods and other research processes from which the conclusions were drawn. The credibility of the conclusions regarding effectiveness or impact was assessed in terms of the methodological rigour of the studies.

## Results

### Study characteristics

Figure 1 describes the process of screening the initial 345 papers selected from the electronic databases and websites down to seven which were finally included for data extraction. Table 3 lists the final set of seven papers, all of which were obtained through database searches.

**Table 3:** Final set of papers including how they were obtained

Author/s and Year	How Located
Aljasir & Alghamdi (2010) [15]	EED^a^
Allen (1996) [12]	EED
Jackson-Pulver (2010) [16]	EED
McDermott et al. (2001) [17]	EED
Roberts-Thomson et al. (2010) [19]	EED
Rowley et al. (2008) [14]	EED
Scrace & Margolis (2009) [18]	EED

^a^*EED* is an abbreviation for Established Electronic Database

The studies varied markedly in design and methodological rigour (see Table 4). Two studies were cluster randomised controlled trials, one was a case study, two were retrospective analyses (with one being cross-sectional and the other being longitudinal), one was a cohort study using population level data as a comparator, and one was a descriptive cross-sectional design.

**Table 4:** Descriptions of the final set of papers in terms of the type of study and the key findings

Author/s and Year	Type of Study	Outcomes Assessed	Key Findings
Aljasir & Alghamdi (2010)	Descriptive cross-sectional design	• Satisfaction with structure of visiting service • Satisfaction with services provided • Overall satisfaction compared with primary health care centres	• 35.8% rated the location of the clinic as unsatisfactory • 20.5% rated the schedule (1/week) as unsatisfactory • 62.5% indicated the mobile clinic provided a lower quality of service compared with a primary health care centre • 90.9% suggested that the mobile clinic was not dependable in providing health care in the area
Allen (1996)	Case study	• Number of clients accessing the service • Reasons for accessing the service • Cost comparison between Outreach Service and Hospital Service	• 59 clients accessed physiotherapy appointments in the first 4 months • 30 clients attended for long-standing musculoskeletal pain syndromes, 29 attended for acute musculoskeletal pain/injuries • $7 to $10 for a hospital occasion of service (15 min direct client contact) compared with $36 for the outreach service
Jackson-Pulver et al. (2010)	Retrospective cross-sectional audit of dental service client records	• Level of dental volunteer involvement, client attendance, and treatment provision • Perceptions of stakeholders regarding practical arrangements, care provided, and future directions	• The program met a pressing need (eliminated a 2 year waiting list) • Enhanced workforce development • Continuity of care was important to virtually all respondents • Cross-cultural relationships were fostered
McDermott et al. (2001)	Unblinded Cluster RCT	• Weight, blood pressure, eye and foot care, serum lipid levels, glucose monitoring and control, urinary albumin to creatinine ratio, serum creatinine levels • Administration of recommended vaccines • Hospitalisation in previous 12 months	• A greater improvement in most measures over the 12 months in the intervention sites • At follow-up, those in the intervention groups were 40% less likely to be hospitalised for a diabetes-related condition than those in control group
Roberts-Thomson et al. (2010)	Cluster RCT	• Oral health promotion activities in the community • Personal oral health practices of children	• No significant differences in the uptake of community level oral health promotion activities between the intervention and control communities at the two year follow-up • No significant difference on the clinical measures between children in the intervention and control groups
Rowley et al. (2008)	Cohort study with population level data as a comparator	• Mortality from all causes and from cardiovascular disease • Hospitalisation with cardiovascular disease coded as a primary cause of admission	• Mortality significantly lower than that of the NT Indigenous population • Hospitalisation with CVD as a primary cause was 13/1000 person-years for the cohort, compared with 33/1000 person-years for the NT Indigenous population.
Scrace & Margolis (2009)	Retrospective longitudinal report comparing historical controls with a dedicated fly-in/fly-out primary care skin cancer outreach clinic	• Skin cancer diagnosis and management	• An increase in the number of lesions removed per year • Four-fold increase in melanoma detection

There was variability in context and service models. Four studies had some sort of comparison group. One study was conducted in Saudi Arabia and the rest in Australia. One service was a mobile, remotely based primary health care service [14]. The remainder were visiting services from major population hubs. The studies covered medical, dental, and non-medical health services.

### Data synthesis

The studies described mixed results – some effective, some not (see Table 4). In the Saudi Arabian study, for example, although participants were satisfied with some aspects of the service, many of them were dissatisfied with the clinic location and the service schedule [15]. A range of different outcomes related to service effectiveness were evident in the studies. Within the studies reviewed there were reported reductions in waiting lists [16], improvements in diabetes management [17], lower mortality and reduced hospitalisations compared with the general population [14], and improved melanoma detection [18]. One of the cluster randomized controlled trials (RCTs) found no significant differences between the intervention and control groups [19].

It is important to note that while each of these seven studies was evaluating a visiting service, the emphasis was generally on the *service itself* rather than the fact that it was *visiting* or *periodic* in the delivery of that service*.* That is, there were no studies comparing a service delivered by resident health professionals compared with the same service delivered by visiting health professionals. Given that the services being provided in these evaluations were, in many ways, standard evidence-based practices and in some cases were additional services, it should come as no surprise that they led to beneficial outcomes. While it will always remain important to establish that services are, in fact, as effective as we expect them to be, and have the desired impact, it is crucial to understand how these evidence-based practices can be delivered most effectively and have maximum positive impact in remote settings where populations are insufficient to support resident services. For that reason, it is the *visiting* component rather than the *service* that needs to be examined more closely.

A number of studies addressed some aspects of the visiting form of delivery of the service. Aljasir and Alghamdi [15] reported that many people were dissatisfied with the visiting service. For example, one in five of the people who were interviewed were dissatisfied with the once per week timing of the service, suggesting that this was insufficient availability to meet patient needs for primary health care. Jackson-Pulver et al. [16], on the other hand, reported that the service being evaluated in their study had met a pressing need and that continuity of care was important and had been addressed in the design of the service (see Table 4). Rowley et al. [14] offered a rationale for a community controlled mobile service that enabled the population to live in a desired decentralised fashion.

## Discussion

In order to obtain the best value and most effective impact from visiting services, decision-makers and service funders require rigorous empirical evidence of what works best in which context. Unfortunately, as this systematic review shows, such evidence is scant. From the sparse literature available, it is not possible to answer our research question. This systematic review demonstrates that more rigorous evaluation of visiting services, their impact, effectiveness, and cost is required, and in particular, the specific impact of the ‘visiting’ modality requires examination. Given the investment in visiting services and the urgency in reducing the disparity in health outcomes between people in remote versus urban locations, it is surprising that so little attention has been given to assessing the effectiveness or impact of visiting services.

The limitations of systematic review methodology in answering some of the questions that are most important to rural and remote health should also be acknowledged. It has been suggested for almost ten years that a more flexible approach to systematic reviews should be adopted in rural and remote health settings [20]. For this reason we relaxed the inclusion and exclusion criteria that we developed and we searched a large number of websites to particularly focus on “grey” literature. In fact, our systematic review has highlighted one of the important issues in rural and remote health which is the lack of available data to inform policy development in important areas.

In the absence of knowledge about effectiveness or impact, health professionals, funders, policy-makers, and evaluators might benefit from considering a number of important principles [2]. The principles offered here have been derived from a synthesis of information obtained from the available literature, the current systematic review, and the authors’ expert knowledge and experience (see Table 5). The first principle relates to the justification of the service and addresses the concern mentioned in the introduction that visiting services are being used in some communities which have populations large enough to support a resident team of primary health care professionals. The second principle focuses on the scheduling of the service and the extent to which the timing of the service is commensurate with the needs of the community. For example, Aljasir and Alghamdi [15] described dissatisfaction relating to service scheduling only once per week. The third principle relates to co-ordination, which refers to the extent to which visiting services are integrated with each other but also linked with resident primary health care services. The fourth principle, scope, addresses whether or not the visiting service is sufficiently comprehensive and targeted to meet the prioritised needs of the community. The importance of the fifth principle (continuity), was highlighted by Jackson-Pulver et al., [16] who reported that virtually all respondents in their study emphasised the importance of continuity of care which had been successfully addressed through continuity of non-dentist staffing. The sixth principle, support, highlights the importance of reciprocity with the visiting health professionals supporting resident staff and resident staff supporting the visiting professionals. Review, the seventh principle, ensures that the effectiveness of the visiting service is monitored, evaluated, and improved. While our research identified a range of different outcome measures related to service effectiveness reported in the literature, future research in this field would ideally report on a suite of measures which assess effectiveness according to each of the identified key principles for providing visiting services. Table five summarises how papers addressed or didn’t address these principles.

**Table 5:** Analysis of papers addressing proposed key principles for effective visiting services

Key Principles	Papers						
	Aljasir & Alghamdi	Allen	Jackson-Pulver et al.	McDermott et al.	Roberts-Thomson et al.	Rowley et al.	Scrace & Margolis
Justification	Service established to improve equity and access	Described community consultations	Inability to attract a resident dentist	Not mentioned	Not mentioned	A “degree of unmet need for medical treatment”; mobile service enables population to stay on traditional homelands	Describes a known higher rate of skin cancer in remote areas
Scheduling	Expressed dissatisfaction relating to service scheduling only once per week.	Not mentioned	Not mentioned although a Steering Committee was established	Not mentioned	Not mentioned	Mentions regular outreach visits “at the direction of the Health Council”	Mentions regularly scheduled visits but not how the schedules are derived
Co-ordination	No resident service and no mention of other services	Describes the presence of existing health infrastructure as a major advantage	Not mentioned although a Steering Committee was established	Not mentioned	Not mentioned	Not mentioned	Not mentioned
Scope	No mention of scope but study participants dissatisfied with range of services	Physiotherapy, occupational therapy, and social work	Dental service	Diabetes outreach service	Oral health	Not mentioned	Skin cancer clinic
Continuity	Not mentioned	Described as essential	Important to “virtually all respondents” & maintained in project design	Not mentioned	Not mentioned	Not mentioned	Mentions continuity of care as an ideal but not achieved in this study
Support	No resident team	Not mentioned	Integration within a primary health care setting described as important	Mentions that this is likely to be important	Integrating oral health care into broader primary care activities described as important	Not mentioned	Describes harnessing local resource to promote the service
Review	The study is an example of the type of review that should occur. No mention made of this occurring routinely.	Not mentioned	This study is an evaluation of the service but no mention made of this occurring routinely.	This study is an evaluation of the service but no mention made of this occurring routinely.	This study is an evaluation of the service but no mention made of this occurring routinely.	Follow up of previous population-based surveys	Not mentioned

## Conclusion

Visiting services are an important component of the delivery of primary health care services to isolated remote and rural communities. In order to ensure residents of remote communities are receiving the best possible primary health care it is important that evaluations of these visiting services are conducted systematically and routinely. Moreover, it is essential that, in addition to evaluating the effectiveness of the service, the appropriateness and efficiency of the visiting modality is also assessed. It is only through careful and comprehensive examination of both the visiting aspects *and* the service aspect that we will be able to make the most astute and informed evidence-based decisions regarding the way in which the health needs and priorities of residents of remote locations can be adequately addressed.

## Abbreviations

FIFO: fly in/fly out
HIC: high income country
LMIC: low-income and middle-income countries
RCT: randomized controlled trial

## References

[1] Australian Institute of Health and Welfare. Rural, regional and remote health: indicators of health status and determinants of health. Rural Health Series no. 9. Cat. no. PHE 97. Canberra: AIHW; 2008. http://www.aihw.gov.au/WorkArea/DownloadAsset.aspx?id=6442459831. Accessed 25 Nov 2016.

[2] Carey TA, Sirett D, Wakerman J, Russell D, Humphreys JS. What principles should guide visiting primary health care services in rural and remote communities? Lessons from a systematic review. BMC Hlth Serv Rsch Under review. 10.1111/ajr.12425 29845693

[3] De Roodenbeke E, Lucas S, Rouzaut A, Bana E (2011). Outreach services as a strategy to increase access to health workers in remote and rural areas.

[4] Australian Government Productivity Commission. Australia’s Health Workforce Commonwealth of Australia: Melbourne; 2005. http://www.pc.gov.au/inquiries/completed/health-workforce/report/healthworkforce.pdf. Accessed 25 Nov 2016.

[5] Wakerman J, Curry R, McEldowney R. Fly in/fly out health services: the panacea or the problem? Rural and Remote Health (Internet). 2012; 12: 2268. http://www.rrh.org.au/publishedarticles/article_print_2268.pdf. Accessed 25 Nov 2016. 22794666

[6] Guerin P, Guerin B (2009). Social effects of fly-in-fly-out and drive-in-drive-out services for remote indigenous communities. Aus Comm Psych.

[7] House of Representatives Standing Committee on Regional Australia. Cancer of the bush or salvation for our cities? Fly-in, fly-out and drive-in, drive-out workforce practices in Regional Australia. Canberra: Parliament of Australia; 2013. file:///C:/Users/care0089/AppData/Local/Downloads/http---www.aphref.aph.gov.au-house-committee-ra-fifodido-report-fullreport.pdf. Accessed 25 Nov 2016.

[8] Battersby MW (2005). Health reform through coordinated care: SA HealthPlus. BMJ.

[9] Wagner EH, Austin BT, Von Korff M (1996). Organizing care for patients with chronic illness. Milbank Q.

[10] Birks M, Mills J, Francis K (2010). Models of health service delivery in remote or isolated areas of Queensland: a multiple case study. Aus J Adv Nurs.

[11] Battye KM, McTaggart K. Development of a model for sustainable delivery of outreach allied health services to remote north-West Queensland, Australia. Rural and Remote Health (Internet) 2003; 3: 194. http://www.rrh.org.au/articles/subviewnew.asp?ArticleID=194. Accessed 25 Nov 2016. 15882095

[12] Allen O (1996). Anthill and other injuries: a case for mobile allied health teams to remote Australia. Aus J Rur Hlth.

[13] Wilkinson L, Beattie N (2006). Enhancing Allied Health Services to Rural and Remote Indigenous Communities: Phase 1.

[14] Rowley KG, O’Dea K, Anderson I, McDermott R, Saraswati K, Tilmouth R, Roberts I, Fitz J, Wang Z, Jenkins A, Best JD, Wang Z, Brown A (2010). Lower than expected morbidity and mortality for an Australian aboriginal population: 10 year follow-up in a decentralised community. MJA..

[15] Aljasir B, Alghamdi MS (2010). Patient satisfaction with mobile clinic services in a remote rural area of Saudi Arabia. Eastern Med Hlth J.

[16] Jackson-Pulver L, Fitzpatrick S, Ritchie J, Norrie M (2010). Filling the gap: an evaluation of a voluntary dental program within an aboriginal and Torres Strait islander community controlled primary health service. Aboriginal & Islander Hlth Work J.

[17] McDermott RA, Schmidt BA, Sinha A, Mills P (2001). Improving diabetes care in the primary healthcare setting: a randomised cluster trial in remote indigenous communities. MJA.

[18] Scrace M, Margolis SA. The Royal Flying Doctor Service primary care skin cancer clinic: A pilot program for remote Australia. Rur Rem Hlth (Internet). 2009; 9: 1048. Available: http://researchonline.jcu.edu.au/5327/. Accessed 27 Nov 2016. 19239334

[19] Roberts-Thomson KF, Slade GD, Bailie RS, Endean C, Simmons B, Leach AJ, Raye I, Morris PS (2010). A comprehensive approach to health promotion for the reduction of dental caries in remote indigenous Australian children: a clustered randomised controlled trial. Int Dent J.

[20] Humphreys JS, Kuipers P, Wakerman J, Wells R, Jones JA, Kinsman LD (2009). How far can systematic reviews inform policy development for “wicked” rural health service problems?. Aus Hlth Rev.

